# Vaccination Coverage with Selected Vaccines and Exemption Rates Among Children in Kindergarten — United States, 2019–20 School Year

**DOI:** 10.15585/mmwr.mm7003a2

**Published:** 2021-01-22

**Authors:** Ranee Seither, Michael T. McGill, Jennifer L. Kriss, Jenelle L. Mellerson, Caitlin Loretan, Kendra Driver, Cynthia L. Knighton, Carla L. Black

**Affiliations:** ^1^Immunization Services Division, National Center for Immunization and Respiratory Disease, CDC; ^2^Certified Technical Experts, Inc., Montgomery, Alabama; ^3^Association of Schools and Programs of Public Health Fellowship, Washington, DC;^ 4^Oak Ridge Institute for Science and Education, Oak Ridge, Tennessee.

State and local school vaccination requirements serve to protect students against vaccine-preventable diseases (*1*). This report summarizes data collected by state and local immunization programs* on vaccination coverage among children in kindergarten (kindergartners) in 48 states, exemptions for kindergartners in 49 states, and provisional enrollment and grace period status for kindergartners in 28 states for the 2019–20 school year, which was more than halfway completed when most schools moved to virtual learning in the spring because of the coronavirus 2019 (COVID-19) pandemic. Nationally, vaccination coverage^†^ was 94.9% for the state-required number of doses of diphtheria and tetanus toxoids, and acellular pertussis vaccine (DTaP); 95.2% for 2 doses of measles, mumps, and rubella vaccine (MMR); and 94.8% for the state-required number of varicella vaccine doses. Although 2.5% of kindergartners had an exemption from at least one vaccine,^§^ another 2.3% were not up to date for MMR and did not have a vaccine exemption. Schools and immunization programs can work together to ensure that undervaccinated students are caught up on vaccinations in preparation for returning to in-person learning. This follow-up is especially important in the current school year, in which undervaccination is likely higher because of disruptions in vaccination during the ongoing COVID-19 pandemic (*2*–*4*).

To meet state and local school entry requirements, parents and guardians submit children’s vaccination records or exemption forms to schools, or schools obtain records from state immunization information systems. Federally funded immunization programs work with departments of education, school nurses, and other school personnel to assess vaccination and exemption status of children, typically those aged 4–6 years, enrolled in public and private kindergartens and to report unweighted counts, aggregated by school type, to CDC via a web-based questionnaire in the Secure Access Management System.^¶^ CDC uses these data to produce state- and national-level estimates of vaccination coverage (*5*). During the 2019–20 school year, 48 states reported coverage for all state-required vaccines among public school kindergartners, and 47 states reported on private school kindergartners.** Forty-nine states reported exemption data among public school kindergartners, and 48 states reported on private school kindergartners.** This report provides data on overall national and median vaccination coverage for the state-required number of doses of DTaP, MMR, and varicella vaccine. Hepatitis B and poliovirus vaccination coverage data, which are not included in this report, are available at SchoolVaxView (*6*). Twenty-eight states reported data on kindergartners who, at the time of assessment, were attending school under a grace period (attendance without proof of complete vaccination or exemption during a set interval) or provisional enrollment (school attendance while completing a catch-up vaccination schedule). Coverage and exemptions from U.S. territories and associated states are presented; however, national estimates, medians, and summary measures include only U.S. states.

Vaccination coverage and exemption estimates were adjusted according to survey type and response rates.^††^ National estimates measure coverage and exemptions among all kindergartners, and medians measure the midpoint of state-level coverage regardless of population size. Reported estimates for the 2019–20 school year are based on 3,675,882 kindergartners surveyed for vaccination coverage, 3,914,961 for exemptions, and 2,955,220 for grace period and provisional enrollment among the 4,025,574 children reported as enrolled in kindergarten by immunization programs for 49 states.^§§^ Potentially achievable coverage with MMR, defined as the sum of the percentage of children who are up to date with 2 doses of MMR and those with no documented vaccination exemption but who are not up to date, was calculated for each state. Nonexempt students include those provisionally enrolled, in a grace period, or otherwise without documentation of vaccination. SAS (version 9.4; SAS Institute Inc.) was used for all analyses.

Vaccination assessments varied by immunization program because of differences in states’ required vaccines and number of doses, vaccines assessed, methods of data collection, and data reported (Supplementary Table 1, https://stacks.cdc.gov/view/cdc/100473). The majority of states reported kindergartners as up to date for a given vaccine if they had received all doses of that vaccine required for school entry.^¶¶^ Seven states*** reported kindergartners as up-to-date for any given vaccine only if they had received all doses of all vaccines required for school entry.

Nationally, 2-dose MMR coverage was 95.2% (range = ≥86.6% [Alabama] to ≥99.1% [Mississippi]). Coverage of ≥95% was reported by 20 states and coverage of <90% by three states (Table). DTaP coverage was 94.9% (range = 84.0% [Indiana] to ≥99.1% [Mississippi]), with 20 states reporting coverage of ≥95%, and three states reporting <90% coverage. Coverage with 2 doses (or 1 dose, as required) of varicella vaccine was 94.8% (range = ≥86.6% [Alabama] to ≥99.1% [Mississippi]), with 21 states reporting coverage ≥95%, and four states reporting <90% coverage.

**TABLE:** Estimated* coverage^†^ with measles, mumps, and rubella vaccine (MMR), diphtheria and tetanus toxoids and acellular pertussis vaccine (DTaP), and varicella vaccines, grace period/provisional enrollment,^§^ and any exemption^¶^ among children enrolled in kindergarten, by immunization program — United States, territories, and associated states, 2019–20 school year

Immunization program	Kindergarten population**	No. (%) surveyed^††^	Coverage (%)			Grace period/Provisional enrollment (%)	Any exemption (%)	Percentage point change in any exemption, 2018 to 2019
			MMR^§§ ^2 doses	DTaP^¶¶ ^5 doses	Varicella*** 2 doses			
**National estimate^†††^**	**4,025,574**	**3,675,882 (91.3)**	**95.2**	**94.9**	**94.8**	**1.6**	**2.5**	**—**
**Median^†††^**	**NA**	**NA**	**94.6**	**94.4**	**94.6**	**1.6**	**2.7**	**0.1**
Alabama^§§§^	59,477	56,416 (94.9)	≥86.6	≥86.6	≥86.6	NP	1.2	0.4
Alaska^§§§,¶¶¶^	10,381	8,580 (82.7)	NR	NR	NR	NR	5.9	−1.2
Arizona****	83,976	82,848 (98.7)	92.8	92.6	95.3	NR	5.5	−0.5
Arkansas^††††^	39,510	37,997 (96.2)	94.3	93.2	93.9	456 (1.2)	1.9	0.1
California^††††^	566,155	554,250 (97.9)	96.5	96.2	96.1	8,262 (1.5)	0.8	0.2
Colorado^§§§§^	69,088	67,876 (98.2)	91.1	92.8	90.1	500 (0.7)	4.9	—
Connecticut^§§§,¶¶¶¶^	38,888	38,888 (100.0)	96.2	96.2	95.9	NP	2.5	−0.2
Delaware^§§§^	NR	NR	NR	NR	NR	NR	NR	NA
District of Columbia^§§§^	NR	NR	NR	NR	NR	NR	NR	NA
Florida^§§§,¶¶¶¶,^*****	228,298	228,298 (100.0)	≥93.5	≥93.5	≥93.5	6,737 (3.0)	3.4	0.2
Georgia^§§§,¶¶¶¶^	130,102	130,102 (100.0)	≥93.1	≥93.1	≥93.1	292 (0.2)	3.0	0.5
Hawaii^§§§^	15,695	1,403 (8.9)	89.7	91.1	91.8	0 (<0.1)	6.1	1.7
Idaho	23,301	22,950 (98.5)	89.1	89.0	88.5	373 (1.6)	7.6	−0.1
Illinois^§§§,¶¶¶¶^	145,891	145,891 (100.0)	96.6	96.5	96.4	925 (0.6)	2.0	0.2
Indiana^§§§^	88,253	57,968 (65.7)	94.4	84.0­	94.0	NR	2.2	0.9
Iowa^§§§,¶¶¶¶^	40,812	40,812 (100.0)	≥93.2	≥93.2	≥93.2	1,255 (3.1)	2.5	0.1
Kansas^§§§,††††,†††††^	37,865	12,996 (34.3)	90.4	90.0	89.6	NR	2.1	—
Kentucky^§§§,††††,^*****	59,233	55,031 (92.9)	93.1	93.3	92.5	NR	1.8	0.4
Louisiana^¶¶¶¶^	59,685	59,685 (100.0)	95.6	97.2	95.0	NP	1.5	0.3
Maine	13,450	13,395 (99.6)	94.1	94.1	96.2	NR	5.9	−0.3
Maryland^,§§§,††††^	72,443	71,225 (98.3)	97.9	98.2	97.5	NR	1.4	−0.1
Massachusetts^§§§,¶¶¶¶,††††^	66,756	66,756 (100.0)	97.3	97.2	97.0	NP	1.3	−0.1
Michigan^¶¶¶¶^	120,565	120,565 (100.0)	94.8	94.7	94.4	798 (0.7)	4.4	−0.1
Minnesota^§§§§,^*****	71,223	70,284 (98.7)	92.6	92.3	92.0	NR	3.8	0.1
Mississippi^§§§,^****,^¶¶¶¶,^	37,870	37,870 (100.0)	≥99.1	≥99.1	≥99.1	17 (<0.1)	0.2	0.1
Missouri^§§§§,¶¶¶¶^	72,324	72,324 (100.0)	94.6	94.5	94.2	NR	2.7	—
Montana^§§§,¶¶¶¶^	12,501	12,501 (100.0)	93.6	93.2	93.2	231 (1.8)	4.3	−0.2
Nebraska^§§§,††††^	26,893	26,012 (96.7)	96.3	96.9	95.6	440 (1.6)	2.2	0.1
Nevada^§§§^	37,724	37,678 (99.9)	95.4	94.0	94.6	896 (2.4)	4.0	0.7
New Hampshire^§§§,¶¶¶¶^	12,447	12,447 (100.0)	≥91.5	≥91.5	≥91.5	561 (4.5)	3.1	−0.2
New Jersey^¶¶¶¶,¶¶¶^	107,900	107,900 (100.0)	≥95.9	≥95.9	≥95.9	958 (0.9)	2.6	0.1
New Mexico^§§§^	23,087	23,087 (100.0)	97.0	96.7	96.7	369 (1.6)	1.5	—
New York (incl. New York City)^§§§,^****	234,165	234,031 (99.9)	98.6	97.8	98.1	3,827 (1.6)	0.1	−1.2
New York City^§§§,^****	96,581	96,447 (99.9)	98.1	97.3	97.7	846 (0.9)	0.1	−0.6
North Carolina ^§§§, ††††,^*****	124,548	121,835 (97.8)	95.5	95.5	95.3	1,499 (1.2)	1.7	0.1
North Dakota	10,587	10,536 (99.5)	94.8	94.4	94.8	NR	3.9	−0.4
Ohio	139,103	137,441 (98.8)	92.4	92.3	91.9	8,515 (6.1)	2.8	−0.1
Oklahoma	55,348	47,374 (85.6)	93.0	93.9	96.9	NR	2.7	0.1
Oregon^††††,¶¶¶¶^	45,959	45,959 (100.0)	93.4	92.6	94.6	NR	7.1	−0.6
Pennsylvania	140,197	138,573 (98.8)	96.6	96.8	96.3	3,085 (2.2)	3.0	0.1
Rhode Island^§§§,††††,^*****	11,219	11,054 (98.5)	97.7	97.4	97.0	NR	1.3	—
South Carolina^§§§^	65,938	18,104 (27.5)	95.0	95.2	94.5	174 (0.3)	2.6	—
South Dakota^§§§^	12,367	12,337 (99.8)	96.0	95.9	95.2	NR	2.7	0.1
Tennessee^§§§,††††,¶¶¶¶^	80,595	80,595 (100.0)	96.8	96.4	96.5	1,529 (1.9)	2.0	0.1
Texas (including Houston)^ ††††,^*****	398,680	397,093 (99.6)	96.9	96.6	96.4	5,507 (1.4)	2.5	0.1
Houston^††††,^*****	38,868	38,655 (99.5)	96.3	96.4	95.2	415 (1.1)	1.5	—
Utah^¶¶¶¶^	49,208	49,208 (100.0)	92.7	92.2	92.4	1,144 (2.3)	5.4	−0.3
Vermont^§§§,¶¶¶¶^	6,293	6,293 (100.0)	94.5	94.1	93.9	262 (4.2)	3.7	−1.0
Virginia^§§§,†††††^	99,399	1,200 (1.2)	94.6	97.6	93.3	NR	1.7	—
Washington*****	87,757	80,623 (91.9)	94.4	92.8	92.7	1,234 (1.4)	5.7	0.7
West Virginia^§§§,^****^,§§§§§^	17,114	8,481 (49.6)	98.2	98.8	97.8	16 (0.1)	0.1	−0.7
Wisconsin^††††,^*****^,†††††^	67,391	1,777 (2.6)	92.8	94.5	91.6	68 (0.1)	5.7	−0.2
Wyoming^¶¶¶¶^	7,913	7,913 (100.0)	94.5	94.4	85.6	152 (1.9)	3.5	0.6
**Territories and associated states**								
American Samoa^§§§,¶¶¶¶,§§§§§^	781	781 (100.0)	91.9	71.4	21.0	NP	—	NA
Federated States of Micronesia^¶¶¶¶^	1,532	1,532 (100.0)	90.7	76.8	NReq	NR	—	—
Guam^§§§^	2,513	2,492 (99.2)	93.7	92.9	NReq	NR	0.1	—
Marshall Islands^§§§,^****^,¶¶¶¶^	1,115	1,115 (100.0)	92.0	90.3	NReq	NR	—	—
Northern Mariana Islands^¶¶¶¶^	895	895 (100.0)	95.3	97.3	95.0	NR	—	—
Palau^¶¶¶¶,¶¶¶¶¶^	273	273 (100.0)	90.1	90.1	NReq	NR	—	—
Puerto Rico	26,980	1,266 (4.7)	93.5	89.9	93.0	NR	2.0	0.4
U.S. Virgin Islands	NR	NR	NR	NR	NR	NR	NR	NA

**Abbreviations:** NA = not available; NP = no grace period/provisional policy; NR = not reported to CDC; NReq = not required for school entry.

* Estimates are adjusted for nonresponse and weighted for sampling where appropriate.

^†^ Estimates based on a completed vaccine series (i.e., not vaccine-specific) use the “≥” symbol. Coverage might include history of disease or laboratory evidence of immunity.

^§^ A grace period is a set number of days during which a student can be enrolled and attend school without proof of complete vaccination or exemption. Provisional enrollment allows a student without complete vaccination or exemption to attend school while completing a catch-up vaccination schedule. In states with one or both of these policies, the estimates represent the number of kindergartners within a grace period, provisionally enrolled, or some combination of these categories.

^¶^ Exemptions, grace period/provisional enrollment, and vaccination coverage status might not be mutually exclusive. Some children enrolled under a grace period/provisional enrollment might be exempt from one or more vaccinations, while children with exemptions might be fully vaccinated with one or more required vaccines.

** The kindergarten population is an approximation provided by each program.

^††^ The number surveyed represents the number of kindergartners surveyed for vaccination coverage. For Alaska, this number represents the number surveyed for exemptions as coverage was not reported. The national total excludes the 10,381 kindergartners from Alaska. Exemption estimates are based on 34,011 kindergartners for Kansas, 65,938 kindergartners for South Carolina, 97,236 kindergartners for Virginia, and 67,391 kindergartners for Wisconsin.

^§§^ Most states require 2 doses of MMR; Alaska, New Jersey, and Oregon require 2 doses of measles, 1 dose of mumps, and 1 dose of rubella vaccines. California, Georgia, New York, New York City, North Carolina, and Virginia require 2 doses of measles and mumps and 1 dose of rubella vaccines. Iowa requires 2 doses of measles and 2 doses of rubella vaccines.

^¶¶^ Pertussis vaccination coverage might include some diphtheria, tetanus toxoids, and pertussis vaccine (DTP) vaccinations if administered in another country or by a vaccination provider who continued to use DTP after 2000. Most states require 5 doses of DTaP for school entry, or 4 doses if the fourth dose was received on or after the fourth birthday; Illinois, Maryland, Virginia, and Wisconsin require 4 doses; Nebraska requires 3 doses. The reported coverage estimates represent the percentage of kindergartners with the state-required number of DTaP doses, except for Kentucky, which requires ≥5 but reports ≥4 doses of DTaP.

*** Most states require 2 doses of varicella vaccine for school entry; Alabama, Arizona, Hawaii, Maine, New Jersey, Oklahoma, and Oregon require 1 dose. Reporting of varicella vaccination status for kindergartners with a history of varicella disease varied within and among states; some were reported as vaccinated against varicella and others as medically exempt.

^†††^ National coverage estimates and medians calculated from data from 48 states (i.e., do not include Alaska, Delaware, and the District of Columbia). National grace period/provisional enrollment estimates and medians were calculated from data from 28 states that have either a grace period or a provisional enrollment policy and that reported relevant data to CDC. National exemption estimates and medians were calculated from data from 49 states (i.e., do not include Delaware and the District of Columbia). Other jurisdictions excluded were Houston, New York City, American Samoa, Guam, Marshall Islands, Federated States of Micronesia, North Mariana Islands, Palau, Puerto Rico, or U.S. Virgin Islands. Data reported from 3,675,882 kindergartners assessed for coverage, 3,914,961 for exemptions, and 2,955,220 for grace period/provisional enrollment. Estimates represent rates for populations of 4,015,193; 4,025,574; and 3,056,534 kindergartners for coverage, exemptions, and grace period/provisional enrollment, respectively.

^§§§^ Philosophical exemptions were not allowed.

^¶¶¶^ Alaska did not report kindergarten vaccination coverage because of problems with data collection. Vaccination coverage among children aged 6 years in VacTrAK, Alaska's Immunization Information System, was 75.5% for MMR, 85.1 for DTaP, and 73.0 for varicella vaccine.

**** Religious exemptions were not allowed.

^†††† ^Counted some or all vaccine doses received regardless of Advisory Committee on Immunization Practices—recommended age and time interval; vaccination coverage rates reported might be higher than those calculated using only valid doses.

^§§§§^ Program did not report the number of children with exemptions, but instead reported the number of exemptions for each vaccine, which could count some children more than once. Lower bounds of the percentage of children with any exemptions were estimated using the individual vaccines with the highest number of exemptions.

^¶¶¶¶^ The proportion surveyed likely was <100% but is reported as 100% based on incomplete information about the actual current enrollment.

***** Did not include some types of schools, such as online schools or those located in military bases or correctional facilities, or tribal lands.

^†††††^ Kindergarten vaccination coverage data were collected from a sample, and exemption data were collected from a census of kindergartners.

^§§§§§^ Reported public school data only.

^¶¶¶¶¶^ For Palau, estimates represent coverage among children in first grade.

The percentage of kindergartners with an exemption from one or more required vaccines (not limited to MMR, DTaP, and varicella vaccines) remained unchanged from the 2018–19 school year at 2.5% (range = 0.1% [New York and West Virginia] to 7.6% [Idaho]) (Table). Nationally, 0.3% of kindergartners had a medical exemption, and 2.2% had a nonmedical exemption (Supplementary Table 2, https://stacks.cdc.gov/view/cdc/100473). Only 95.2% of kindergartners were up to date with MMR; 2.5% had an exemption from at least one vaccine,^§^ and another 2.3% were not up to date with MMR and did not have a vaccine exemption (Table).

The percentage of kindergartners attending school within a grace period or provisionally enrolled among the 28 states reporting these data was 1.6% (range = <0.1% [Hawaii and Mississippi] to 6.1% [Ohio]) (Table). Of the 28 states with MMR coverage <95%, 24 states could potentially achieve ≥95% MMR coverage if all nonexempt kindergartners, many of whom were within a grace period or provisionally enrolled, were vaccinated (Figure 1). Among the 30 states reporting a decrease in the percentage of kindergartners who were not up to date for MMR and did not have an exemption in 2019–2020 compared with 2018–2019, an increase of MMR coverage in 2019–2020 was also reported by 26 states (Figure 2). In three states with MMR coverage <95% in 2018–2019 (Illinois, North Carolina, and South Carolina), coverage increased to ≥95% in 2019–2020.

**FIGURE 1:**
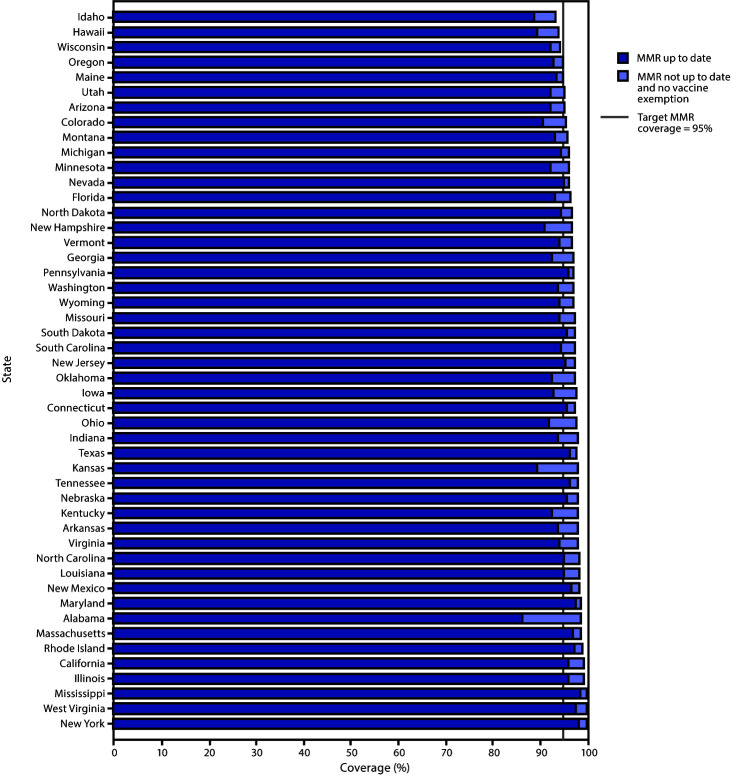
Potentially achievable coverage*^,†,§ ^with measles, mumps, and rubella vaccine (MMR) among kindergartners, by state — 48 states, 2019–20 school year * States are ranked from lowest to highest potentially achievable coverage. Potentially achievable coverage is estimated as the sum of the percentage of students with up-to-date MMR and the percentage of students without up-to-date MMR and without a documented vaccine exemption. ^†^ The exemptions used to calculate the potential increase in MMR coverage for Arizona, Arkansas, Colorado, Idaho, Illinois, Maine, Massachusetts, Michigan, Minnesota, Missouri, Nebraska, New York, North Carolina, North Dakota, Ohio, Oklahoma, Oregon, Rhode Island, Texas, Utah, Vermont, Wisconsin, and Wyoming are the number of children with exemptions specifically for MMR vaccine. For all other states, numbers are based on an exemption to any vaccine. ^§^ Alaska, Delaware, and the District of Columbia did not report kindergarten vaccination coverage for the 2019–20 school year and are excluded from this analysis.

**FIGURE 2:**
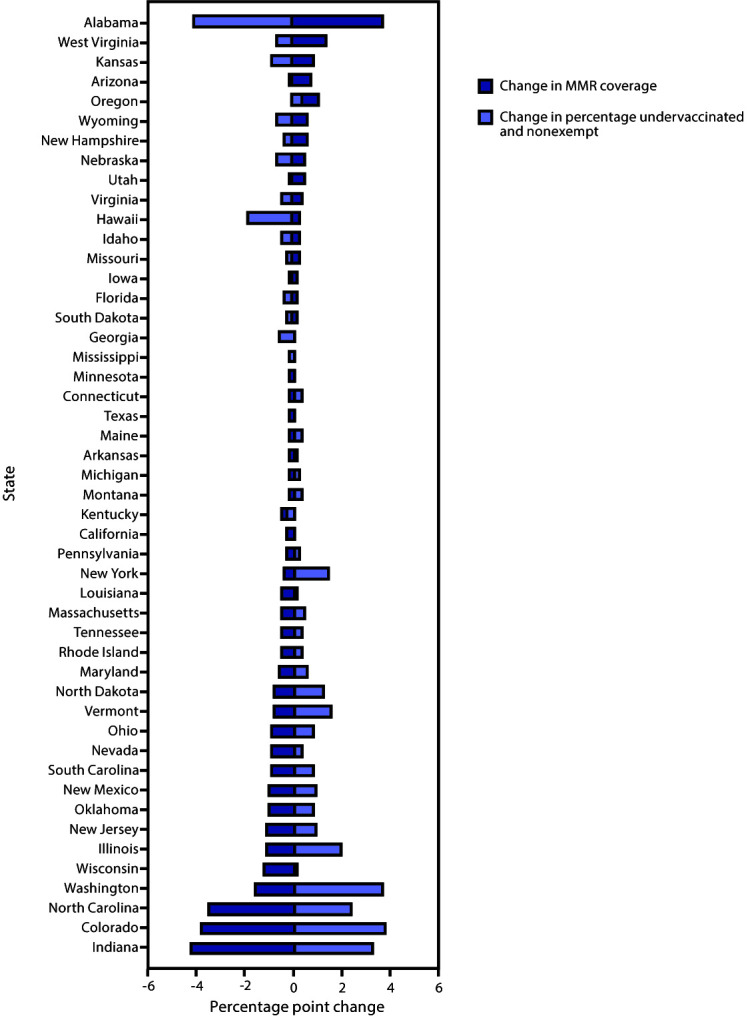
Change in percentage of kindergartners who are fully vaccinated with measles, mumps, and rubella vaccine (MMR) and in the percentage who are undervaccinated and nonexempt,*^,†,§^ by state — 48 states, 2018–19 to 2019–20 school years * States are ranked from greatest decrease to highest increase in the percentage of kindergartners who are undervaccinated and nonexempt. The exemptions used to calculate the MMR not up to date and no documented vaccine exemptions for Arizona, Arkansas, Colorado, Idaho, Illinois, Maine, Massachusetts, Michigan, Minnesota, Missouri, Nebraska, New York, North Carolina, North Dakota, Ohio, Oklahoma, Oregon, Rhode Island, Texas, Utah, Vermont, Wisconsin, and Wyoming are the number of children with exemptions specifically for MMR vaccine. For all other states, numbers are based on an exemption to any vaccine. ^†^ Alaska, Delaware, and the District of Columbia did not report kindergarten vaccination coverage for the 2019–20 school year and are excluded from this analysis. ^§^ California, Michigan, New Jersey, New York, Oregon, Pennsylvania, Texas, and Washington experienced >15 cases of measles during the 2018–2019 outbreak.

## Discussion

The purpose of vaccination assessment is to identify populations at risk and aid in taking programmatic steps to increase vaccination coverage. Although the COVID-19 pandemic led to late, truncated, or incomplete assessment of kindergarten vaccination status in the 2019–20 school year compared with the 2018–19 school year in some states (*7*), most student vaccinations would have already occurred before the start of the 2019–20 school year and would not have been affected by the pandemic. National coverage among kindergartners remained approximately 95% (*5*) for MMR, DTaP, and varicella vaccines. However, coverage and exemption rates varied by state. Measles outbreaks that affected school-aged children across multiple states during the 2018–19 school year underscore the importance of both school vaccination requirements for preventing disease spread and school coverage assessments to identify pockets of low coverage (*8*). Among eight states with measles outbreaks of ≥15 cases during the 2018–19 school year (*8*), six reported increases in MMR coverage during 2019–2020. Increases in some states were likely attributable to changes in state laws eliminating nonmedical vaccination exemptions (*9*), and vaccination campaigns in response to the outbreaks could also have contributed to the increases in MMR coverage.

The overall percentage of children with an exemption remained at approximately 2.5%; children with exemptions represent a small proportion of kindergartners nationally and in most states. In 25 states, the number of nonexempt undervaccinated kindergartners equaled or exceeded the number of those with exemptions. In many states, nonexempt undervaccinated students are attending school in a grace period or are provisionally enrolled. Follow-up with undervaccinated students can increase vaccination coverage in this group.

Twenty-six states successfully increased MMR coverage by reducing the number of nonexempt students who are not up to date, with three states (Illinois, North Carolina, and South Carolina) reaching coverage of ≥95%. Some states have implemented policies and activities focused on improving coverage. In Colorado, MMR coverage increased from 87.4% in 2018–2019 to 91.1% in 2019–2020. This was accomplished by prioritizing high MMR coverage. In addition to providing technical assistance, media toolkits, strategies, and local kindergarten MMR data and targets, the state health department furnished lists of elementary schools with low coverage to local public health agencies, which implemented community-specific strategies. These included digital media campaigns aimed at parents, vaccination reminder/recall, efforts to improve school compliance, outbreak tabletop exercises with schools, and incentives to families (Diana Herrero, Colorado Department of Public Health and Environment, personal communication, November 13, 2020). Almost all states could achieve ≥95% MMR coverage if nonexempt undervaccinated children were vaccinated according to local and state vaccination policies.

The findings in this report are subject to at least six limitations. First, comparability is limited because of variation in states’ requirements, data collection methods, exemptions allowed, and definitions of grace period and provisional enrollment. Second, representativeness might be negatively affected because of data collection methods that missed some schools or students or occurred at different times. Third, results might be underestimated or overestimated because of incomplete documentation. Fourth, national coverage estimates include only 48 of 50 states but use lower bound estimates for seven states; exemption estimates include 49 states but use lower bound estimates for three states; and grace period or provisional enrollment estimates include only 28 states for the 2019–20 school year. Fifth, estimates of potentially achievable MMR coverage are approximations and are underestimated for states that do not report vaccine-specific exemptions (*5*,*7*). Finally, because of the COVID-19 pandemic, schools were closed, and state and local health department staff members were deployed to response activities, limiting the quantity and quality of student vaccination data collected and reported to CDC (CDC, unpublished data, 2020).

Based on measurements from other data sources, CDC expects that the COVID-19 pandemic has already reduced actual vaccination coverage of kindergarten-aged children through reduced appointment availability at providers’ offices, parents delaying preventive health care visits, and other barriers to vaccination, and that those disruptions will reduce kindergarten vaccination coverage in the 2020–21 school year (*2*–*4*). In addition, schools in many states began the 2020–21 school year remotely and might not have enforced the usual vaccination policies. Providers, schools, and immunization programs will need to increase follow-up with undervaccinated students and find ways to overcome pandemic-related barriers to maintain the high level of vaccination coverage necessary to continue protecting school-aged children, their family members, and communities from vaccine-preventable diseases during virtual learning and as schools return to in-person instruction. Jurisdictions should provide resources as appropriate, such as guidance to parents about the importance of maintaining preventive care during the pandemic, lists of immunization providers in the area for children who are unable to be vaccinated by their usual health care provider, or special vaccination clinics at schools or health departments.

**Summary**

**What is already known about this topic?**

State immunization programs conduct annual kindergarten vaccination assessments to monitor school-entry vaccination coverage with all state-required vaccines.

**What is added by this report?**

For the 2019–20 school year, national coverage was approximately 95% for diphtheria and tetanus toxoids, and acellular pertussis; measles, mumps, and rubella; and varicella vaccines. The national exemption rate remained low at 2.5%.

**What are the implications for public health practice?**

Disruptions caused by the COVID-19 pandemic are expected to reduce vaccination coverage in the 2020–21 school year. Increased follow-up of undervaccinated students is needed from schools and immunization programs to maintain the high vaccination coverage necessary to protect students in preparation for schools returning to in-person learning.

## Footnotes

* Federally funded immunization programs are located in the 50 states and the District of Columbia (DC), five other cities or counties, and eight U.S territories and freely associated states (territories). Two cities reported data to CDC, which were also included in data by their state, to calculate medians and national estimates. Immunization programs in U.S. territories reported vaccination coverage and exemptions to CDC; however, these data were not included in overall national calculations.

† National and median vaccination coverage was determined using estimates for 48 states; Alaska, Delaware, and the District of Columbia did not report school coverage data because of problems with data collection. Data from cities were included with their state data. Data from territories were not included in national and median calculations.

§ National and median exemption rates were determined using estimates for 49 states; Colorado, Minnesota, and Missouri did not collect information on the number of kindergartners with an exemption but instead reported the number of exemptions for each vaccine, which could count some children more than once. For these states, the percentage of kindergartners exempt from the vaccine with the highest number of exemptions (the lower bound of the potential range of exemptions) was included in the national and median exemption rates. Delaware and the District of Columbia did not report school vaccine exemption data because of problems with data collection. Data from cities were included with their state data. Data from territories were not included in national estimates.

¶ Assessment date varied by state and area. Four states assess on the first day of school; 14 states assess by December 31; 16 states assess by some other date, ranging from 30 days after admission to April 30; 15 states assess on a rolling basis.

** Ten states reported coverage and exemption data for at least some homeschooled kindergartners as follows: California included data for students who attend virtual partial or full charter schools with some or all online instruction and students receiving individualized education program services because they are medically unable to attend school in public school data, and data for homeschools with six or more students in private school data. Kansas included data for students educated through public virtual schools in the public school data. New Mexico included all homeschooled students in public school data. North Dakota reported some homeschool data separately. Oregon reported data for students enrolled in exclusively online homeschool programs separately; online students of otherwise traditional public schools were included in the public school data. Pennsylvania included all homeschooled students in their public school data. South Carolina and Wisconsin included homeschooled students in their public or private school data if the students also attended classes, extracurricular activities, or had other contact with a school. Vermont included homeschooled students in their public and private school data if the students were enrolled in one or more classes at a school. Wyoming reported homeschooled students in their public school data if the students also attend classes or extracurricular activities at a public school.

†† Most immunization programs that used census or voluntary response provided CDC with data aggregated at the state or local (city or territory) level. Coverage and exemption data based on a census or voluntary response were adjusted for nonresponse using the inverse of the response rate, stratified by school type (public, private, and homeschool, where available). Programs that used complex sample surveys provided CDC with deidentified data aggregated at the school or county level for weighted analysis. Weights were calculated to account for sample design and adjusted for nonresponse for data collected through complex sample design wherever possible.

§§ The totals reported here are the summations of the kindergartners surveyed among programs reporting data for coverage, exemptions, grace periods, and provisional enrollment. Data from cities and territories were not included in these totals.

¶¶ All states required 2 doses of a measles-containing vaccine. Local DTaP requirements varied: Nebraska required 3 doses, four states (Illinois, Maryland, Virginia, and Wisconsin) required 4 doses, and all other states required 5 doses, unless the fouth dose was administered on or after the fourth birthday. The reported coverage estimates represent the percentage of kindergartners with the state-required number of DTaP doses, except for Kentucky, which required 5 doses of DTaP by age 5 years but reported 4-dose coverage for kindergartners. Seven states required 1 dose of varicella vaccine; 44 states required 2 doses.

*** Alabama, Florida, Georgia, Iowa, Mississippi, New Hampshire, and New Jersey did not assess coverage for individual vaccines, but instead considered kindergartners up to date only if they had received all doses of all vaccines required for school entry. For these states, estimates are shown with the “≥” symbol.
