# *Erratum:* Vol. 70, No. 3

**DOI:** 10.15585/mmwr.mm7013a5

**Published:** 2021-04-02

In the report “Vaccination Coverage with Selected Vaccines and Exemption Rates Among Children in Kindergarten — United States, 2019–20 School Year,” on page 77, in the Table, for the row for Kansas, in the columns labeled “MMR 2 doses,” “DTaP 5 doses,” and “Varicella 2 doses,” the percentages should have been **90.0%**, **89.7%**, and **89.1%**, respectively.

